# Low-Protein Diet during Lactation and Maternal Metabolism in Rats

**DOI:** 10.5402/2011/876502

**Published:** 2010-10-20

**Authors:** Vera L. Moretto, Marcia O. Ballen, Talita S. S. Gonçalves, Nair H. Kawashita, Luiz F. Stoppiglia, Roberto V. Veloso, Márcia Q. Latorraca, Maria Salete F. Martins, Maria Helena G. Gomes-da-Silva

**Affiliations:** ^1^Curso de Pós Graduação em Ciências da Saúde, Faculdade de Ciências Médicas, Universidade Federal de Mato Grosso (UFMT), 78060-900 Cuiabá, MT, Brazil; ^2^Programa de Iniciação Científica, Fundação de Apoio à Pesquisa do Estado de Mato Grosso (FAPEMAT), 78050-970 Cuiabá, MT, Brazil; ^3^Departamento de Química, ICET, UFMT, 78060-900 Cuiabá, MT, Brazil; ^4^Departamento de Alimentos e Nutrição, Faculdade de Nutrição (FANUT), Universidade Federal de Mato Grosso (UFMT), Avenida Fernando Correa da Costa, 2367. Bairro Boa Esperança, 78060-900 Cuiabá, MT, Brazil

## Abstract

Some metabolic alterations were evaluated in Wistar rats which received control or low-protein (17%; 6%) diets, from the pregnancy until the end of lactation: control non-lactating (CNL), lactating (CL), low-protein non-lactating (LPNL) and lactating (LPL) groups. Despite the increased food intake by LPL dams, both LP groups reduced protein intake and final body mass was lower in LPL. Higher serum glucose occurred in both LP groups. Lactation induced lower insulin and glucagon levels, but these were reduced by LP diet. Prolactin levels rose in lactating, but were impaired in LPL, followed by losses of mammary gland (MAG) mass and, a fall in serum leptin in lactating dams. Lipid content also reduced in MAG and gonadal white adipose tissue of lactating and, in LPL, contributed to a decreased daily milk production, and consequent impairment of body mass gain by LPL pups. Liver mass, lipid content and ATP-citrate enzyme activity were increased by lactation, but malic enzyme and lipid: glycogen ratio elevated only in LPL. *Conclusion.* LP diet reduced the development of MAG and prolactin secretion which compromised milk production and pups growth. Moreover, this diet enhanced the store of lipid to glycogen ratio and suggests a higher risk of fatty liver development.

## 1. Introduction

Lipids are the major source of energy for most tissues during periods of negative energy balance, but in some circumstances they can have pathological effects [1]. Triacylglycerol (TAG) is stored in various adipose tissue depots of body, but if blood nonesterified fatty acid (NEFA) levels are elevated for prolonged periods, as it may occur during lactation or obesity, TAG can accumulate in other tissues including liver and muscle cells and can have pathological consequences such as the development of ketosis [2], type 2 diabetes [3], or nonalcoholic fatty liver [4]. During lactation, liver, adipose tissue, and mammary gland (MAG) are the major sites of fatty acid metabolism and are able to synthesize the *in vivo* fatty acids *de novo* and esterify them to TAG [1]. 

Lactation comprises a catabolic mode of adipose tissue metabolism, with markedly reduced fatty acid synthesis/esterification and low-lipoprotein lipase activity [5, 6]. By contrast, lipogenesis is increased in MAG, ensuring a preferential uptake of TAG precursors for milk fat production [7]. In this phase, MAG becomes the most active site of lipogenesis, exceeding by 4-fold the liver [8].

The liver has a more complex role in lipid metabolism than adipose or mammary tissue, taken up NEFA from the blood and either oxidizing them to CO_2_ or ketones (ketoacetate and *β*-hydroxybutyrate), which are released into the blood for use elsewhere in the body, or esterifying fatty acids to TAG and phospholipids, which are then secreted into the blood as lipoproteins including very low density lipoproteins (VLDL). In lactation, several mechanisms promote the oxidation of fatty acids in the liver [2, 6], which are related to a fall in serum insulin accompanied by a rise in glucagon and also in NEFA [9]. Despite the fall in the serum insulin to glucagon ratio and other mechanisms that operate to promote fatty acid oxidation, fatty acid esterification is also increased in the liver during lactation. This is primarily due to the substantial increase in NEFA uptake but, in addition, the activities of some esterification enzymes are also increased [10]. The physiological purpose of the TAG accumulation in the liver is not clear. It may be a default of a system in which NEFA uptake by the liver is determined by supply rather than need, reflecting the role of the liver in regulating the nutrient composition of the blood [11, 12].

According to Choi et al. [13], the intrauterine growth retardation caused by protein deficiency of the mother plays an important role in the adult life development of “fatty liver” or fat accumulation within liver. It also increased risk of adult metabolic syndrome, clustering cardiovascular risk factors such as diabetes, hypertension, dyslipidemia, and obesity [14]. This liver lipid accumulation is a feature seen in protein-calorie malnutrition such as kwashiorkor [4].

In our laboratory, we observed low-protein dams on the 14th day of lactation lower serum insulin levels and this study showed that the maternal metabolic adaptation to hypoinsulinaemia resulted in higher insulin sensitivity, enhanced carcass fat deposition, hyperleptinaemia, and hypophagia [15]. The aim of the present study was to evaluate some metabolic alterations on maternal metabolism in low-protein rats in the latest phase of lactation.

## 2. Methods

### 2.1. Animals and Diets

The experiment was formally approved by the institutional ethical committee and followed the COBEA guidelines (Brazilian College of Experimental Animal) adopted by Mato Grosso Federal University (UFMT) [16]. Female Wistar rats (90d) were supplied by the animal central care facility of the UFMT, Cuiaba, Brazil. Mating was performed by housing females with males overnight and pregnancy was confirmed by the presence of sperm in vaginal smears. Virgin and pregnant females were separated and maintained from the first day of pregnancy until the 18th day of lactation with isocaloric diets containing 60 g protein/kg (low-protein diet) or 170 g protein/kg (control diet) as described by Ferreira et al. [15].

Spontaneous delivery took place the 22nd day of pregnancy and after which, at 3 days of age, large litters were reduced to eight pups, ensuring a standard litter size per mother, that were weighed three times a week until the end of experimental period. Lactating and non-lactating rats were divided in four groups and evaluated from the first day until the 18th day of lactation, as follows: control non-lactating (CNL) and lactating (CL), low-protein non-lactating (LPNL), and lactating (LPL), with free access to food and water. The control non-lactating and low-protein non-lactating groups were formed by virgin rats. They were kept under standard lighting conditions (12 h light/dark cycle) at a 24 ± 1°C of temperature. Food intake was evaluated at same times of animal weighting. 

Milk production in each group was estimated as described by do Carmo et al. [17] as the difference between weights of the offspring soon after suckling and after 24 h fasting, at the 16th day of lactation. The differences were taken as the amount of milk suckled from the dams.

### 2.2. Sample Collection and Analyses

At the end of the experimental period (18th day of lactation) the rats were euthanized with CO_2_ and the blood was collected after decapitation. Serum was obtained by centrifugation and aliquots were stored at −80°C. Serum glucose was measured by the glucose oxidase method (Accu-chek, Roche Diagnostics, Germany), total protein by the biuret modified method [18] and albumin by the green bromocresol method [19]. Serum hormone concentrations were analyzed by ELISA assay cross-reaction kits for rats: insulin (Linco Research, USA), prolactin (Alpco Diagnostics, USA), leptin (Antigenix American Inc., USA), and glucagon (Wako, USA). 

Gonadal white adipose tissue (GON), liver, mammary gland (MAG), and carcass (CARC) were quickly removed after euthanasia for fresh weight determination (g) and kept at −80°C until its use for dosages. Liver fragments were excised to determination of glycogen content [20]. The fat content in the tissues was measured according to Folch method [21], and values were expressed as mg of lipid/100 mg of tissue. Carcasses lipid was analyzed as described by Oller de Nascimento and Williamson [22] and values were expressed as mg of lipid/100 mg of carcass.

### 2.3. Measurements of Enzymes Activities

Hepatic enzymes activities were measured by the following methods: glucose-6-phosphate dehydrogenase (G6PDH) was assayed as described by Lee [23], malic enzyme (ME) by the method of Ochoa [24] modified by Hsu and Lardy [25]. Both assays were performed by measuring the rate of formation of NADP. ATP-citrate lyase (ATP-cit) was assayed as described by Srere [26], measuring the rate of oxidation of NADH. Absorbance was taken each 30 s at 340 nm. Enzymes activities were expressed as nmol NADH · mg of protei*n* ^−1^ *·* mi*n* ^−1^. Protein concentration was determined as described by Lowry [27].

### 2.4. Statistical Analyses

Results were expressed as mean ± SEM for the number of rats indicated. Levene's test for homogeneity of variances was initially used to determine whether data complied with the assumptions of parametric analysis of variance. When necessary, data were log-transformed to correct for variance in heterogeneity or nonnormality. All data were subsequently analyzed by two-way ANOVA (nutritional status and physiologic status) followed by Tukey-HSD test for individual differences among groups. Different superscript letters were employed to mark statistical differences. Student's *t* test was used to compare two groups. *P* < .05 indicated statistical significance. All statistical comparisons were done using the Statistics Software Package (Statsoft, Tulsa, OK, USA).

## 3. Results

Absolute food intake was similar in low-protein lactating dams compared to controls, but the increases were found in both LPL and LPNL when normalizing it by percentage of body mass. Protein intake was reduced in absolute and relative terms in low-protein groups. By the end of experimental period, LPL group significantly reduced the final body mass (Table 1). 

Serum protein concentration was significantly lower in LPL, but albumin levels were reduced in both low-protein groups (LPNL and LPL). In an opposite way, serum glucose values of these rats were higher than the controls (Table 2). 

 Lactation lowered serum insulin and glucagon levels, markedly in LPL dams, being that we found insulinemia 3.5 times lower than in CL. On the other hand, low-protein diet reduced both insulin and glucagon levels when compared to controls. And, although the lowest ratios of insulin/glucose and insulin/glucagon in lactating groups (LPL, CL) the low-protein diet reduced the insulin/glucose ratio. Lactation induced rise in serum prolactin levels in both LPL and CL, but it was impaired in low-protein dams and, a fall in serum leptin levels was observed in both control and low-protein lactating dams (Table 2).

**Figure 1:**
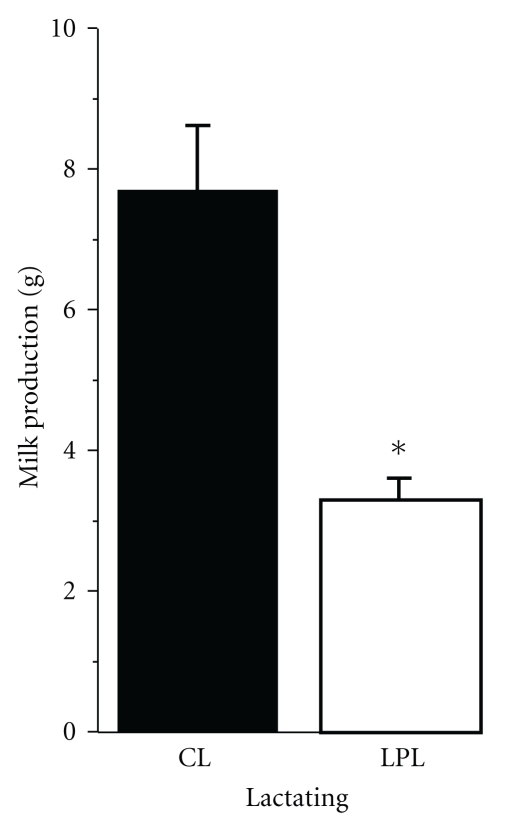
Daily milk production by control (CL) and low-protein (LPL) lactating. Bars are means ± SEM, *n* = 7 mothers. *Statistically different from control lactating (Student's *t*-test; *P* < .05).

**Figure 2:**
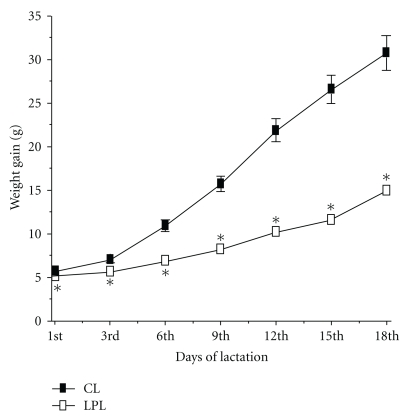
Weight gain of pups weaned by control (CL) and low-protein (LPL) lactating. Values are means ± SEM; *n* = 7. *P < .05 relative to CL (Student's *t*-test). Pups of control and low-protein mothers were weighed each 3 days after birth until the 18th day of lactation.

By this period, LPL dams lost some relative mass of mammary glands, whereas control dams retained their MAG weights. Lipid content also reduced in MAG of control and low-protein rats (Table 3). Daily milk production was decreased in LPL group too, according to Figure 1, with consequent impairment of body mass gain in LPL pups (Figure 2).

Other lipid-providing tissue such as GON was reduced in both lactating groups (LPL and CL) (Table 3).

Table 4 shows the liver parameters analyzed. In lactating rats, the mass (g) and the lipid content (%) were increased in this tissue as compared to non-lactating ones (in absolute and relative values), although the absolute mass had decrease in both low-protein groups. Glycogen content did not differ between groups, but the lipid-to-glycogen ratio in the liver elevated in LPL rats. Following these parameters, the liver lipogenic enzymes G6PDH and ATP-cit activities were markedly increased by lactation, and in low-protein dams (LPL) higher levels of malic enzyme activity was observed. 

## 4. Discussion

Despite the highest initial body mass and relative food intake observed, the lactating low-protein dams reached the lowest values of final body mass and decrease of serum albumin concentration was observed in low-protein animals. This is a well-described consequence of scarce nitrogen stores for milk-protein synthesis in low-protein malnourishment [5, 8, 28]. The lactation-induced loss of mammary gland weight in protein-restricted dams (with lowered milk production) then argues for a general modification of nutrient metabolism, where milk synthesis is impaired at the glandular level.

According to Dewey [28], a suitable protein supply in maternal diet is necessary to enhance milk production and allows a positive increase in milk's protein balance. Zambrano et al. [29] described that offspring whose mothers have restricted protein diet during lactation weighed less than those whose mothers were on the control diet. Additionally, Park et al. [14] indicated that poor nutrition in early life, especially protein restriction, causes long-lasting changes in mitochondria, and this change is more evident in the liver and skeletal muscle, that may contribute to the development of insulin resistance in later life. Choi et al. [13] demonstrated the occurrence of structural changes in the liver and, important changes in lipid metabolism in rats submitted to 25% of food restriction.

Serum prolactin was 70% lower in low-protein dams, endowing the low MAG weight observed and putting the impaired milk production on a pituitary/hormonal level. Recent studies demonstrated that the actions of prolactin are not confined to the mammary gland [30], but contributes to maternal leptin resistance, increased food intake, and maternal behaviors immediately after parturition [30, 31]. 

Lactation half end is characterized by decreased insulinemia and leptinemia. These low insulin and leptin levels in lactating rats are associated to catabolic processes releasing fatty acids from the adipose tissue, towards to the liver. In lactating low-protein dams the insulinemia reduced in 2-thirds as compared to control lactating, prompting to a higher release of fatty acids. According to Sipols et al. [32], low insulin and leptin concentrations can lead to hyperphagia, which could have contributed to the elevation of food intake verified in both lactating groups [6, 33].

We found increased serum glucose levels and reduced insulinemia in low-protein groups, especially in lactating dams. Moreover, in low-protein lactating rats the insulin/glucagon ratio was 46% lower than in control. As described, a higher fatty acid mobilization from depots is usually observed during lactation, to provide fatty acids towards the mammary gland and other tissues [5, 6]. The lactating groups exhibited a reduction in the mass and lipid content of gonadal adipose tissue on the 18th day of lactation. In our model, since this lipid mobilization did not result in increased milk production, a first place for lipid deposition is the liver.

Both lactating groups exhibited increased mass and lipid content in the liver. Accumulation of fats in the liver during early, but not in late lactation, is usually verified and seldom impairs its functions [12]. The stored lipid-to-glycogen ratio in low-protein lactating group was, however, very higher (3 : 1), and even in control lactating group this parameter was in the limit (2 : 1). Previous studies had shown that when this ratio exceeds about 2 : 1, pathological problems begin to develop [2]. The reason for the pathological effects of high levels of TAG in liver is unclear, but it can relate to prolonged elevation of nonesterified fatty acids (NEFA) levels and/or their CoA esters in the cell [34]. Because lipid homeostasis is mainly dependent on the liver, the underlying lipid deregulation in lactating nutritional restricted rats would be mediated, at least in part, through alterations of liver structure [13, 14].

Additionally, lactation strongly increased the liver lipogenic enzymes in lactating dams, as compared to non-lactating females, especially malic enzyme that elevated only in low-protein dams. Hormonal and nutritional conditions that modify liver lipogenic enzymes activities are described [35, 36], with insulin being stimulatory and glucagon a depressor [37]. Previous studies showed that a high carbohydrate diet stimulates the expression of liver lipogenic enzymes [38] and contributes to elevate plasma TAG levels [39]. In our model, diet protein was replaced by carbohydrate for keeping isocaloric diets. This model did not increase lipogenic enzymes in non-lactating females and even decreased malic enzyme activity both in low-protein non-lactating, as in control lactating. This pattern of alterations is very likely related to the insulin to glucagon ratios observed, reinforcing the hormonal control of liver lipogenesis.

Previous works with this model of protein deficiency in pregnancy and lactation have stated permanent changes in expression of liver enzymes involved in glucose homeostasis [40, 41], increases of liver insulin sensitivity [26, 41] and reduction of glucose tolerance [42]. Such studies explored the offspring physiology but not the dams. 

In this study we observed that low-protein diet during lactation decreased the development of mammary gland and prolactin secretion which resulted in lower milk production and impaired pups growth. Lactation enhanced liver mass, lipid content and lipogenic enzymes activities. Moreover, this diet enhanced the store of lipid to glycogen ratio and suggests a higher risk of fatty liver development.

## Figures and Tables

**Table 1:** Absolute (g) and relative (g/100 g body mass) food intake, protein intake, initial and final body mass of non-lactating and lactating rats maintained with control (CNL and CL) or low-protein (LPNL and LPL) diets.

	GROUPS			
	CNL	CL	LPNL	LPL
Absolute food intake	293 ± 18^b^	573 ± 26^a^	276 ± 11^b^	308 ± 9^b^
Relative food intake	92 ± 1^c^	100 ± 1^b^	94 ± 1^c^	116 ± 4^a^
Absolute protein intake	50 ± 8^b^	97 ± 4^a^	16.5 ± 0.6^c^	19.3 ± 0.9^c^
Relative protein intake	15.5 ± 0.1^b^	17.0 ± 0.2^a^	5.60 ± 0.04^d^	7.0 ± 0.2^c^
Initial body mass	268 ± 4	404 ± 10*	255 ± 2^#^	362 ± 16^∗#^
Final body mass	300 ± 7^b^	330 ± 8^c^	287 ± 5^b^	249 ± 10^a^

CNL, CL, LPNL groups = 7; LPL group = 8 rats. Values are means ± SEM.

*Statistical difference related to non-lactating rats (Two-Way ANOVA; *P* < .05).

^#^Statistical difference related to control rats (Two-Way ANOVA; *P* < .05).

Different letters indicate statistical differences (Tukey HSD test; *P* < .05).

**Table 2:** Serum concentration of total protein, albumin, glucose, insulin, glucagon, leptin, prolactin, insulin/glucose, and insulin/glucagon ratios of non-lactating and lactating rats fed control (CNL and CL) or low-protein (LPNL and LPL) diets.

	GROUPS			
	CNL	CL	LPNL	LPL
Total protein (g/dL)	4.9 ± 0.4^b^	5.2 ± 0.4^b^	4.9 ± 0.6^b^	3.4 ± 0.3^a^
Albumin (g/dL)	3.1 ± 0.1	2.7 ± 0.2	2.2 ± 0.3^#^	1.8 ± 0.2^#^
Glucose (mg/dL)	109 ± 2	108 ± 6	124 ± 4^#^	112 ± 4^#^
Insulin (ng/mL)	3.5 ± 0.8	1.7 ± 0.5*	1.8 ± 0.2^#^	0.52 ± 0.08^∗#^
Glucagon (ng/mL)	1.1 ± 0.2	0.6 ± 0.1*	0.6 ± 0.2^#^	0.5 ± 0.1^∗#^
Leptin (ng/mL)	2.5 ± 0.7	1.1 ± 0.2*	3.7 ± 0.6	1.1 ± 0.3*
Prolactin (ng/mL)	27 ± 4^c^	130 ± 4^a^	22 ± 8^c^	65 ± 6^b^
Insulin/glucose molar ratio	102 ± 22	53 ± 18*	47 ± 3^#^	15 ± 2^∗#^
Insulin/glucagon molar ratio	1.4 ± 0.4	1.1 ± 0.2*	1.5 ± 0.2	0.5 ± 0.1*

CNL, CL, LPNL groups = 7; LPL group = 8 rats. Values are means ± SEM.

*Statistical difference related to non-lactating rats (Two-Way ANOVA; *P* < .05).

^#^Statistical difference related to control rats (Two-Way ANOVA; *P* < .05).

Different letters indicate statistical differences (Tukey HSD test; *P* < .05).

**Table 3:** Relative mass (g/100g body mass) and lipid content (g) of tissues and carcass of non-lactating and lactating rats fed a control (CNL and CL) or low-protein (LPNL and LPL) diets.

	GROUPS			
	CNL	CL	LPNL	LPL
	Relative mass of tissues (g/100 g)			

MAG	2.0 ± 0.2^c^	3.2 ± 0.2^a^	3.0 ± 0.3^b^	2.5 ± 0.2^c^
GON	5.4 ± 0.3	4.2 ± 0.3*	6.2 ± 0.5	4.4 ± 0.4*
CARC	72.6 ± 0.8	69.2 ± 0.5*	72.4 ± 0.8	71.4 ± 0.5*

	Lipid content (g/100 g)			

MAG	68 ± 2	21 ± 5.0*	71 ± 1.0	32 ± 7.0*
GON	79.2 ± 3.5	78 ± 2.0*	79.6 ± 0.6	76 ±1.0*
CARC	9.0 ± 1.0	6.4 ± 0.7	8.2 ± 0.9	6.3 ± 1.0

CNL, CL, LPNL groups = 7; LPL group = 8 rats. Values are means ± SEM.

*Statistical difference related to non-lactating rats (Two-Way ANOVA; *P* < .05).

^#^Statistical difference related to control rats (Two-Way ANOVA; *P* < .05).

Different letters indicate statistical differences (Tukey HSD test; *P* < .05).

**Table 4:** Liver mass, glycogen content, lipid content, lipid/glycogen ratio, and lipogenic enzymes activities (nmol · mg of protein^−1^· min^−1^) of non-lactating and lactating rats fed a control (CNL and CL) or low-protein (LPNL and LPL) diets.

	GROUPS			
	CNL	CL	LPNL	LPL
Absolute mass (g)	9.4 ± 0.3	14.7 ± 0.8*	8.9 ± 0.3^#^	12.4 ± 0.7^∗#^
Relative mass (g/100 g)	3.2 ± 0.1	4.5 ± 0.2*	3.1 ± 0.1	5.0 ± 0.2*
Glycogen (mg/100 mg)	3.8 ± 0.3	5.1 ± 0.5	5.2 ± 0.4	4.7 ± 0.8
Lipid (mg/100 mg)	5.3 ± 0.7	9.3 ± 0.7*	5.4 ± 0.8	11.5 ± 0.9*
Lipid/glycogen ratio	1.4 ± 0.2^c^	2.0 ± 0.3^b^	1.1 ± 0.2^c^	3.1 ± 0.3^a^

Lipogenic enzymes activities (nmol · mg of protein^−1^· min^−1^) (*n* = 5)				

G6PDH	148 ± 10^b^	222 ± 23^a^	62 ± 10^c^	217 ± 15^a^
ATP-cit	142 ± 7	239 ± 34*	136 ± 15	221 ± 17*
EM	68 ± 9^b^	80 ± 3^b^	47 ± 3^c^	139 ± 14^a^

CNL, CL, LPNL groups = 7; LPL group = 8 rats. Lipogenic enzymes activities were obtained from 5 animals. Values are means ± SEM.

*Statistical difference related to non-lactating rats (Two-Way ANOVA; *P* < .05).

^#^Statistical difference related to control rats (Two-Way ANOVA; *P* < .05).

Different letters indicate statistical differences (Tukey HSD test; *P* < .05).

## Abbreviations

TAG: Triacylglycerol
NEFA: Nonesterified fatty acid
MAG: Mammary gland
VLDL: Very low density lipoproteins
CNL: Control non-lactating group
CL: Lactating group
LPNL: Low-protein non-lactating group
LPL: Lactating group
GON: Gonadal white adipose tissue
CARC: Carcass
G6PDH: Glucose-6-phosphate dehydrogenase enzyme
ME: Malic enzyme
ATP-cit: ATP-citrate lyase enzyme
ELISA: Enzyme-linked immunosorbent assay
ANOVA: Analysis of variance.
